# The Correlation Between Otitis Media With Effusion and Adenoid Hypertrophy Among Pediatric ‎Patients: A Systematic Review‎

**DOI:** 10.7759/cureus.30985

**Published:** 2022-11-01

**Authors:** Ghadi D Mashat, Hadrian Hoang-Vu Tran, Neway A Urgessa, Prabhitha Geethakumari, Prathima Kampa, Rakesh Parchuri, Renu Bhandari, Ali R Alnasser, Aqsa Akram, Saikat Kar, Fatema Osman, Pousette Hamid

**Affiliations:** 1 Pediatrics, California Institute of Behavioral Neurosciences & Psychology, Fairfield, USA; 2 Internal Medicine, California Institute of Behavioral Neurosciences & Psychology, Fairfield, USA; 3 Research, California Institute of Behavioral Neurosciences & Psychology, Fairfield, USA; 4 Internal Medicine/Family Medicine, California Institute of Behavioral Neurosciences & Psychology, Fairfield, USA; 5 General Surgery, California Institute of Behavioral Neurosciences & Psychology, Fairfield, USA; 6 Internal Medicine, California institute of behavioral neurosciences & psychology, Fairfield, USA; 7 Neurosciences and Psychology, California Institute of Behavioral Neurosciences & Psychology, Fairfield, USA; 8 Neurology, California Institute of Behavioral Neurosciences & Psychology, Fairfield, USA

**Keywords:** otitis media with effusion, pharyngeal tonsil hypertrophy‎, enlarged adenoids, otitis media, adenoid hypertrophy

## Abstract

Otitis media with effusion (OME) affects approximately 80% of children due to the middle ear being flooded with fluids, though with no microbial infection manifestations. Multiple issues can drive recurring pediatric OME, such as environment-based issues, previous medical issues, inherited vulnerability from family, contact time at childcare institutes, passive smoking, and more than three siblings together with atopy or allergic rhinitis. If OME is not promptly addressed, this could eventually result in hearing impairment or loss, with consequent negative repercussions on the child's communicative and behavioral patterns. OME diagnosis within the clinic is possible, with hearing capacity being assessed pre- and post-therapy. Adenoid hypertrophy (AH) represents a typical causative factor for middle-ear conditions, stemming from mechanical or anatomical issues. Consequently, adenoid size is paramount when determining tympanometry types and ear fluids. This systematic review investigated PubMed, Medline, Cochrane Library, and Science Direct databases in order to retrieve knowledge related to this issue, adopting inclusion and exclusion criteria and maintaining review quality through the employment of the Assessment of Multiple Systematic Reviews (AMSTAR), the Newcastle-Ottawa tool, and the Axis scale. This systematic review analyzed a previous review article, six observation-based investigations, and three cross-sectional investigations. Previous randomized controlled trials (RCTs) were not found within previous literature, suggesting such scarcity in this research niche and thus warranting future RCT investigations based on this compelling research niche.

## Introduction and background

Otitis media with effusion (OME) is predominant within pediatric patients since this ailment is typically non-symptomatic; therefore, it is easy to miss [1]. Pediatric OME cases can suffer from hearing losses that affect communication and behavioral traits. Consequently, age-specific audiometry-based hearing assessments should be carried out in such cases, both prior to and following therapy [1]. Tympanic membrane analyses can lead to OME clinical diagnosis, with a pure-tone / impedance-based audiometer platforms enabling objective determination of audio levels [2,3]. Type-B tympanogram is highly reliable as a non-invasive analytical tool for diagnosing OME [2,3].

Multiple issues lead to recurring pediatric OME, particularly during the very early years of life, with predominating etiopathogenesis issues being allergic rhinitis, recurrent upper respiratory tract infections, gastroesophageal reflux, and adenoid hypertrophy (AH) [4]. 

Middle-ear conditions are highly linked to AH, though specific mechanisms for such pathogenesis are still elusive [5,6]. AH could mechanically hinder the Eustachian tube, if this anatomical ventilation tube is blocked, consequently inducing middle-ear negative pressure and driving clinical conditions [5]. Furthermore, microbial pathogens can migrate towards hyperplastic adenoid pad surfaces due to such middle-ear negative pressure [7]. There is a study that demonstrated that adenoid sizes and middle-ear effusion viscosity share important correlations, regardless of the type and timeframe of clinical symptoms [8]. Grade four AH was also linked to exacerbated severity in middle-ear effusion viscosity increases [8].

A separate observation-based investigation carried out at the Split University Hospital Centre included cases having defined designations for adenoidectomy with myringotomy, and subsequent ventilation tube insertion. OME + AH cases were typically diagnosed within the two to five and six to nine age brackets, at an incidence rate of 49.23% and 46.15%, accordingly [9]. 

Swift AH prophylaxis in order to circumvent pediatric OME is still a major challenge. OME remains to be a prevailing pediatric manifestation requiring therapeutic measures. The long-term aim of this systematic review is to ascertain if pediatric AH cases are in increased danger - or otherwise - of incurring OME, in comparison to non-AH pediatric cases.

## Review

Methodology

A systematic review was carried out for previous literature concerning the above-described theme, in line with the Preferred Reporting Items for Systematic Reviews and Meta-Analyses (PRISMA) directives [10].

Databases

Literature searches were carried out within PubMed, Medline, Science direct, and Cochrane Library, dating between 7/10/2017 and 07/10/2022. The Boolean “AND” term was added to a list of search-words, consisting of:

"otitis media", "adenoid hypertrophy", "enlarged adenoids", and "pharyngeal tonsil hypertrophy". A PubMed search was ‎conducted using MeSH (Medical Subject Headings): ((( "Otitis Media/etiology"[Mesh] OR ‎‎"Otitis Media/physiopathology"[Mesh] )) AND ( "Adenoids/abnormalities"[Mesh] OR ‎‎"Adenoids/physiopathology"[Mesh] )) OR "Hypertrophy"[Mesh].

Article Screening and Inclusion / Exclusion Criteria

Duplicates were identified and excluded, following such screenings. All other articles were scrutinized for abstract and article title. Depending upon article quality analyses (incorporating solely-relevant articles reaching > 60% of quality-appraisal criteria), full-text versions of the results sections were assessed. Other inclusion criteria were English-written articles focusing on pediatric patients (< 18 years of age). Exclusion criteria were articles utilizing in vivo investigations and/or incorporating adult patients.

Risk Bias Evaluation

Quality-appraisal applications were employed for identifying previous literature bias within this systematic review. Founded upon a quality-appraisal threshold of > 60%, a total of 10 articles were chosen, as described in Table 1.

**Table 1:** Quality Assessment Tools ‎ AMSTAR: Assessment of Multiple Systematic Reviews

Study Types	Quality Assessment Tools
Systematic reviews and meta-analyses	AMSTAR checklist
Observational studies	Newcastle-Ottawa tool
Cross-sectional study	Axis scale

Results

Research Outcomes

Founded upon MeSH and search-words across four literature repositories, this study scrutinized 88,963 articles, producing 1599 results following the elimination of 470 duplicate articles and also excluding 86,894 ineligible articles. Subsequently, a first screening session filtered the remaining articles according to inclusion and exclusion criteria, with a second screening session for titles / abstracts, leading to an overall of 22 relevant articles.

Ultimately, 10 articles were shortlisted, comprising one previous systematic review, six observational studies, together with three cross-sectional studies.

**Figure 1:**
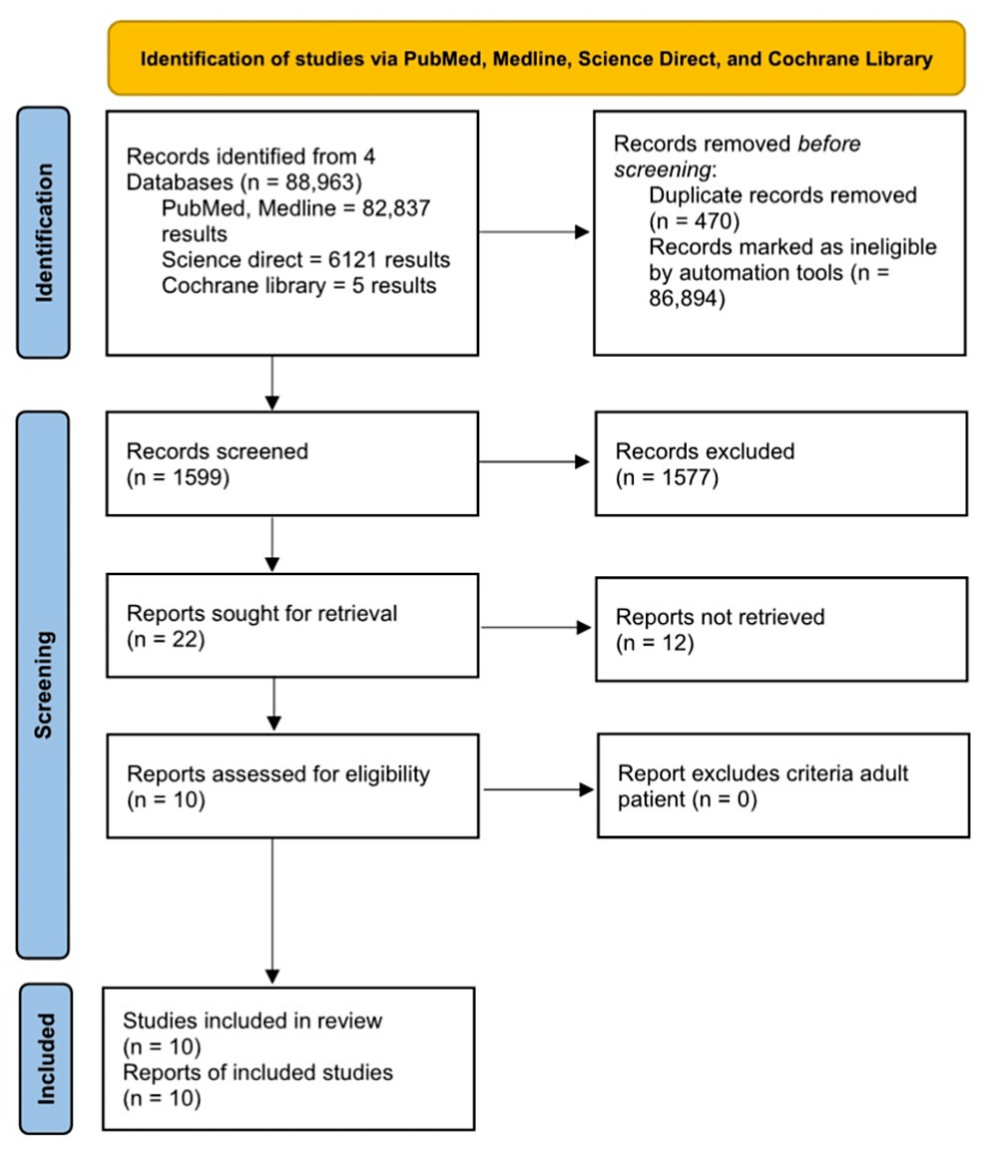
PRISMA Flow Diagram Depicting the Screening Process for this review‎ PRISMA: Preferred Reporting Items for Systematic Reviews and Meta-Analyses

The PRISMA article filtering process for this systematic review is illustrated in Figure 1 [10].

Study Features

Following comprehensive filtering-based selection analyses, 10 previously published articles recognized 3135 clinical cases (Table 2). The overall agreement regarding individual article Results sections scrutinized in this review identified AH to be highly linked to middle-ear conditions.

**Table 2:** Adenoid Hypertrophy Associations in Otitis Media With Effusion Development OME: otitis media with effusion; AH: adenoid hypertrophy; URTIs: upper respiratory tract infections

Author and Year of Publication	purpose	Number of patients	Type of Study	Conclusion
Vanneste et al. 2019 [1]	Providing relevant data regarding OME	-	Systematic review	An otoscope can be used to diagnose OME, and different treatments can be applied
Abdel Tawab et al. 2021 [8]	An analysis of the correlation between AH and tympanometry findings in OME	100	Observational study	OME incidence is strongly correlated with adenoid size
Galić et al. 2022 [9]	A relationship between adenoid size and OME in children	65	Observational study	children with High-grade AH are more likely to have persistent OME, requiring surgical intervention in the event of conservative treatment failure
Niedzielski et al. 2021 [11]	Assessing the quality of life of children with AH from a health-related perspective	101	Observational study	Children suffering from adenoid hypertrophy due to chronic URTIs, leading to decreased quality-of-life.
Songu et al. 2020 [12]	To determine the risks factor of OME in children with AH	539	Observational study	Several factors contribute to OME, including AH, atopy, allergic rhinitis, frequent tonsillitis, attendance at daycare centers, exposure to smoke, and having three or more siblings
Johnston et al. 2019 [13]	An analysis of adenotonsillar and middle-ear microbiota	10	Observational study	Microenvironments in the adenoid do not correlate with those in the middle ear
Sogebi et al. 2021 [14]	Estimate the impact of enlarged adenoids on the development of OME in children	108	Observational study	The prevalence of asymptomatic OME was 29.2% in children with an enlarged adenoid
Bhat et al. 2018 [15]	The relationship between asymptomatic OM and AH	100	Cross-Sectional study	Silent progressive hearing loss can occur due to the neglected symptoms of AH
Saad et al. 2020 [4]	Identify the associated risk factors for recurrent OM with effusion and determine their prevalence	2003	Cross-sectional study	Multi-factorial causes contribute to recurrent OME in children, including AH, rhinitis, recurrent URTIs, together with gastroesophageal reflux
Els et al. 2018 [16]	Prevalence of OME in adenotonsillectomy patients	109	Cross-sectional study	AH does not correlate significantly with OME

Discussion

This systematic review served to ascertain if pediatric cases of AH are correlated to increased risk for incurring OME. Consequently, prior awareness of this correlation will assist in preventing hearing loss within such patients (and consequent communication / behavioral repercussions), by implementing rapid AH diagnosis and prophylaxis.

Overview, Prevalence, and Risk Factors for OME

Uniform approaches for OME theragnostics can be augmented through enhanced knowledge of associations across AH relative physical dimensions and recurring OME incidence rates [9]. One systematic review highlighted pediatric OME cases to be non-symptomatic and challenging to identify, leading to hearing loss and reducing the child’s communication and behavior traits [1]. Multiple pathophysiological factors are linked to OME, such as the inflammatory hypothesis, biofilms, gastroesophageal reflux, and allergies. Often enough, a diagnosis is conducted within the clinical setting, using otoscopy and tympanometry (occasionally). Nasal endoscopy is required on suspecting obstructive AH or unilateral OME. Even though multiple drug therapies exist for treating OME, such approaches are not highly reliable and do not guarantee long-term solutions. Tympanostomy tubes (TT) remain the golden-standard therapy for OME, together with adenoidectomy if required [1]. TT efficacy can be augmented through adenoidectomy. Adenoidectomy is highly indicated in young pediatric AH cases (less than four years of age‎), though could be conducted at older age brackets, should nasal endoscopy identify OME. Prior and post-therapy warrants audiometry assessments (employing age-adapted methodologies). Follow-ups are paramount for such pediatric cases, until OME fully subsides, in order to circumvent the manifestation of complications [1].

Regarding the comparative analysis of the two shortlisted investigations, the first was a cross-sectional study carried out across two tertiary-referral institutes within upper Egypt, aiming to ascertain the incidence rate for recurring OME, together with potential risk variables [4]. Pediatric (< 18 years of age) OM cases were referred to this site. A medical profile was compiled for all enrolled patients, followed by ear, nose, and throat (ENT) assessments. A type-B tympanogram confirmed all OME diagnoses, while otomicroscopic analysis identified air bubbles and fluid presence behind the tympanic membrane. A TT was firstly inserted (bi- and uni-laterally) for first-time-episode cases of OME. Post-surgical clinic follow-up sessions were designated for 10-days post-surgery, with subsequent sessions designated at 90-day intervals, for a total period of 24 months post-first follow-up session. Overall, data was gathered from 2003 cases, consisting of 1016 males (50.7%). Overall cases for OME were 310, consisting of 159 males (51.3%). Within the total study cohort, 15.5% were diagnosed with OME. Such a multi-faceted ailment is comprised of allergic rhinitis, recurrent upper respiratory tract infections, gastroesophageal reflux, AH, and tonsil hypertrophy [4].

The second shortlisted study in this systematic review focused on the issue that AH-diagnosed pediatric cases should have an awareness of crucial risk variables driving OME [12]. This study consisted of the AH + OME Group (n = 110), and the AH + AH Group (n = 429), all undergoing surgery [12]. Datasets were gathered regarding neonatal feeding history, environmental variables (including presence of pets, childcare center attendance rates, and school district), previous medical history (including atopy, allergic rhinitis, apnoea, coughing, tonsillitis within the previous 12 months) [12]. Apart from family history (family size and parents’ educational level), family income and number of siblings were included [12]. This study highlighted no variations existed across cohorts for age and gender [12]. In addition, childcare center attendance, passive smoking, and three siblings, together with dwelling in a habitation comprising four family members, were all high-risk factors for OME, together with atopy and allergic rhinitis [12].

Conversely, one cross-sectional study studied OME incidence rates together with quality-of-life post-adenotonsillectomy among pediatric cases in a hospital within Pretoria, South Africa [16]. Overall, 109 children ‎from two- to 12-year-olds ‎were randomized into cohorts for this investigation, whereby all enrolled patients were exposed to screening through pneumatic otoscopy and tympanometry [16]. As a measure for assessing AH, the adenoid-nasopharyngeal ratio (ANR) was determined across a lateral post-nasal space radiograph [16]. All participants in this study compiled the OM-6 questionnaire, while OME patient participants were evaluated through pure-tone audiometry [16]. OME was markedly linked to atopy, with OME manifestation not correlating with marked statistical significance with AH among 43% of participants [16]. This suggested that OME could be driven by adenotonsillar pathology stemming from biofilm formation, rather than obstructive AH [16]. OME prevailed within 11.9% of bilateral cases and 22.9% of unilateral cases [16]. The OM-6 survey score failed to vary markedly across pediatric cases with / without OME, having a mean total of 1.67 and a mean score of 1.31, respectively [16]. Within such a population cohort, OME had no effect on quality-of-life. Additional investigations are warranted for assessing OM-6's validity within South African pediatric populations [16].

Association of OME and AH

The shortlisted cross-sectional investigation identified in this systematic review was carried out at the Justice K.S. Hegde Hospital in Karnataka, India, and evaluated any associations across non-symptomatic OME and AH within 100 pediatric cases (greater than four years of age) manifesting an adenoid-nasopharyngeal ratio greater than 0.5 [15]. This particular investigation excluded all cases manifesting ontological issues [15]. Within scrutinized cases, 26% were non-symptomatic OME-diagnosed. In addition, greater than 25 decibels (25 dB) in conductive hearing loss was identified within greater than 40% of patients with a bilateral B tympanogram [15]. Impedance audiometry assisted in diagnosing fluid within the middle-ear cavity for all AH cases, thus suggesting that swift medical action is paramount for circumventing complications is essential to avoid potential complications [15].

Within an observation-based investigation scrutinizing 108 children, dataset outcomes highlighted that 49.1% had an age range of ‎one to three years of age ‎[14]. Furthermore, 62.0% of enrolled children were males, weighing 7.8 - 31.0 Kg. Tonsillar enlargement was linked to 63.9% of overall cases [14]. Following from tympanometry datasets, 29.2% of all patient ears were confirmed for OME, with 30.5% of these being unilateral, while 19.4% occurred within the right ear, 11.1% being left ear, and 13.9% were bilateral OME cases [14]. In addition, 29.6% of cases exhibited no bilateral acoustic reflexes, while 42.1% of participants had normal hearing, and the remainder of this cohort experienced differing types of hearing. Interestingly, AH was found to be markedly linked to weight and older age [14].

A separate observation-based study conclude that OME manifestations were associated with AH / tympanometry dataset outcomes, revealing that 100 COME + AH pediatric cases (60 males, 40 females, all aged ≤ 12 years of age), were enrolled in this investigation, according to tympanometry and nasal endoscopic datasets [8]. Gradings for adenoid dimensions were carried out, together with associations according to tympanometry and middle ear fluid typing. Grade four AH exhibited a strongly significant association with mucoid-type middle ear fluid [8]. Furthermore, type B tympanometry and AH grade four were found to be intimately associated with statistical significance. Consequently, adenoid dimensions are considered to be a potential predictor for is a significant predictor tympanometry / middle-ear fluid typing [8].

The third shortlisted observation-based investigation enrolled 65 cases (37 males; 28 females) with confirmed COME + AH manifestations, together with unsuccessful conservative therapy [9]. Individual case histories and analytical results were compiled during pre-diagnostic procedures [9]. Apart from tympanometry and audiometry, a flexible nasal fiberoptic endoscopy was carried out [9]. During endoscopic observations, Cassano grading for adenoid tissue was carried out [9]. A significant level of COME + AH cases were diagnosed among younger-aged cohorts (two to five and six to nine years of age) [9]. A statistically significant outcome was reached for AH grades two and three, suggesting that high-graded AH is paramount for COME development, leading to failures in conservative therapies [9].

In a separate study shortlisted in this review, children (eight to 12 years of age; 54 girls; 47 boys; mean age was 8.62 years) compiled the Child Health Questionnaire-Parent Form 50 (CHQ-PF50) [11]. This investigation demonstrated that no major variations existed across social activity, pain, discomfort, or family cohesion [11]. Peak reductions in well-being were identified within AH cases, mainly affecting behavior, general health concepts, and mental health [11].

Interestingly, Johnston and colleagues postulated that adenoids serve as microbial pathogen reservoirs that drive OM, though previous proof of such a pustulation remained scarce [13]. Their comparative analytical investigation focused on bacterial microbiota within the adenotonsillar and middle-ear cavities of 10 children, though no association between the adenoid micro-environment and the middle-ear cavity was identified in this investigation [13].

According to this systematic review, adenoid hypertrophy is more prevalent in children and neglected symptoms cause asymptomatic and progressive hearing loss. Early intervention must be taken in patients with AH in order to avoid potential complications. A routine checkup after acute otitis media will assist children in preventing hearing loss by educating caregivers regarding risk factors for OME.

Limitations

There were limitations to this systematic review with a shortlist of 10 articles. This study was limited since none of the four databases contained randomized controlled trials (RCTs). This could be partially due to our inclusion criteria, which focused on English-language literature published in the last five years and focused strictly on children under 18 years of age. Due to such stringent inclusion/exclusion criteria, several articles could have been excluded. Moreover, sample size and study timeframes varied across articles such as Abdel et al., which had 100 participants, and Galić et al., which had 65 patients, thus restricting accurate comparative analyses [8,9]. In addition, one study conducted by Vanneste et al. had a quality assessment < 60%‎ though was still included within this systematic review due to its direct relevance to the research niche investigated in this review exercise [1]‎.

## Conclusions

This study aimed to determine whether otitis media is prevalent within children diagnosed with adenoid hypertrophy (AH). We found a correlation between AH and otitis media with effusion (OME), which contributed to silent, progressive hearing loss in children - ultimately impairing child communication and behavior. However, only one study detected that there was no significant correlation between both conditions. High AH grades can contribute to chronic otitis media with effusion (COME) and could lead to conservative therapy failures, while adenoid and middle-ear micro-environments middle were not interlinked. Typically, children with AH should have an adenoidectomy before the age of four years of age, though if nasal endoscopy reveals OME, the procedure can be performed later. Consequently, further randomized controlled trials (RCTs) should be conducted, according to our study conclusions.

## References

[1] Vanneste P, Page C (2019). Otitis media with effusion in children: Pathophysiology, diagnosis, and treatment. A review. J Otol.

[2] Yellon RF, Doyle WJ, Whiteside TL, Diven WF, March AR, Fireman P (1995). Cytokines, immunoglobulins, and bacterial pathogens in middle ear effusions. Arch Otolaryngol Head Neck Surg.

[3] Günel C, Ermişler B, Başak HS (2014). The effect of adenoid hypertrophy on tympanometric findings in children without hearing loss. Kulak Burun Bogaz Ihtis Derg.

[4] Saad K, Abdelmoghny A, Abdel-Raheem YF, Gad EF, Elhoufey A (2021). Prevalence and associated risk factors of recurrent otitis media with effusion in children in Upper Egypt. World J Otorhinolaryngol Head Neck Surg.

[5] Johnston J, Hoggard M, Biswas K, Astudillo-García C, Radcliff FJ, Mahadevan M, Douglas RG (2018). Paired analysis of the microbiota between surface tissue swabs and biopsies from pediatric patients undergoing adenotonsillectomy. Int J Pediatr Otorhinolaryngol.

[6] Abdelhamid AO, Sobhy TS, El-Mehairy HM, Hamid O (2019). Role of antibiotics in post-tonsillectomy morbidities; A systematic review. Int J Pediatr Otorhinolaryngol.

[7] Khoramrooz SS, Mirsalehian A, Emaneini M (2012). Frequency of alloicoccus otitidis, streptococcus pneumoniae, moraxella catarrhalis and haemophilus influenzae in children with otitis media with effusion (OME) in Iranian patients. Auris Nasus Larynx.

[8] Abdel Tawab HM, Tabook SM (2021). Correlation between adenoid hypertrophy, tympanometry findings, and viscosity of middle ear fluid in chronic otitis media with effusion, southern Oman. Ear Nose Throat J.

[9] Galić MZ, Klančnik M (2022). Adenoid size in children with otitis media with effusion. Acta Clin Croat.

[10] Page MJ, McKenzie JE, Bossuyt PM (2021). The PRISMA 2020 statement: an updated guideline for reporting systematic reviews. BMJ.

[11] Niedzielski A, Chmielik LP, Kasprzyk A, Stankiewicz T, Mielnik-Niedzielska G (2021). Health-Related Quality of Life Assessed in Children with Adenoid Hypertrophy. Int J Environ Res Public Health.

[12] Songu M, Islek A, Imre A, Aslan H, Aladag I, Pinar E, Oncel S (2020). Risk factors for otitis media with effusion in children with adenoid hypertrophy. Acta Otorhinolaryngol Ital.

[13] Johnston J, Hoggard M, Biswas K, Astudillo-García C, Radcliff FJ, Mahadevan M, Douglas RG (2019). Pathogen reservoir hypothesis investigated by analyses of the adenotonsillar and middle ear microbiota. Int J Pediatr Otorhinolaryngol.

[14] Sogebi OA, Oyewole EA, Ogunbanwo O (2021). Asymptomatic otitis media with effusion in children with adenoid enlargement. J Natl Med Assoc.

[15] Bhat V, Paraekulam Mani I, Aroor R, Saldanha M, Goutham MK, Pratap D (2019). Association of asymptomatic otitis media with effusion in patients with adenoid hypertrophy. J Otol.

[16] Els T, Olwoch IP (2018). The prevalence and impact of otitis media with effusion in children admitted for adeno-tonsillectomy at Dr George Mukhari Academic Hospital, Pretoria, South Africa. Int J Pediatr Otorhinolaryngol.

